# Procedural Skills Decay in Emergency Medicine: A Scoping Review

**DOI:** 10.7759/cureus.104804

**Published:** 2026-03-07

**Authors:** Kathryn Oskar, Elise Prehoda, Richard Sapp, Xin Qi, Brittany Botticelli, Janice C Palaganas

**Affiliations:** 1 Emergency Medicine, University of Vermont, Burlington, USA; 2 Emergency Medicine, Mass General Brigham, Boston, USA; 3 Emergency Medicine, Massachusetts General Hospital, Harvard Medical School, Boston, USA; 4 Emergency Medicine, Mass General Brigham/Harvard Medical School, Boston, USA; 5 Health Professions Education, MGH Institute of Health Professions, Boston, USA

**Keywords:** emergency medicine physician, emergency medicine procedures, emergency medicine training, procedural skills training, skills decay

## Abstract

While the concept of “skill decay,” or the gradual loss of acquired skills after a period of non-use, has been discussed in the literature, it is not clear how and when this occurs for different procedural skills within the practice of emergency medicine (EM). In this scoping review, we explore the findings and highlight the gaps in the literature about procedural skill decay in EM. Following PRISMA-ScR guidelines, a systematic scoping review with descriptive content analysis was performed. We screened 1349 published abstracts and articles. Thirty-seven (37) articles met the inclusion criteria. Five key themes were identified: (1) limited focus on skill decay as a primary outcome, (2) short and variable timeframes of assessment, (3) limited representation of procedural diversity, (4) heavy reliance on simulation and checklist-based assessments, and (5) sparse data on attending physicians and community-based practice. While there is growing recognition that technical skill decay is a relevant concern in EM, this scoping review reveals a literature base that is heterogeneous in design, limited in range and scope, and still evolving. This review may serve as a call to action for further study on EM procedural skill decay starting points and inform future study design. More rigorous, longitudinal, and generalizable research is required to establish clear, evidence-based practices for maintaining procedural competency across the continuum of emergency physician training and practice.

## Introduction and background

Skill decay, also referred to as “skill fade” or “deskilling”, is the gradual loss of acquired skills that occurs through infrequent practice or extended periods of skill nonuse [1]. In the practice of emergency medicine (EM), where critical, lifesaving procedures may be performed infrequently, the risk of skill decay is not benign, as medical errors and procedural complications pose risks to patient safety [2]. Although there is a recognized concern around procedural skill decay and a broad understanding of skill maintenance as a distinct and important phase of the learning curve for any skill in the scope of emergency medicine [3], little is known about the warranty on skills after initial competence has been reached. In this scoping review, we sought to answer the following research question: For emergency medicine attending and resident trainee physicians, what are the findings and gaps in the literature about technical or procedural skill decay?

The concern around and experience of procedural skills decay among emergency physicians has been reported as high as 93% and 80%, respectively, in one single-site academic needs assessment [4]. Additionally, a large survey of pediatric emergency medicine physicians found that over 90% of respondents felt it was very or extremely important to maintain procedural competency, but only 49% felt clinical practice provided enough opportunity to maintain skills [5]. For high acuity low occurrence (HALO) procedures, supplemental training beyond regular clinical shift experience is needed due to the low frequency of performance reported both in pediatric and adult ED patient populations and in both academic and community ED settings [6-17].

Although preventing procedural skill decay in EM physicians by a standardized process may improve patient-centered outcomes; physician self-esteem, well-being, and confidence; and improve health system-related measures, including reducing costs and complication rates, a survey of Accreditation Council for Graduate Medical Education (ACGME)-accredited emergency medicine programs in 2020 demonstrated most programs lack a formalized method to assess and maintain attending physician procedural competency [18]. Understanding the most effective training methods, including optimal frequency of skill performance in clinical practice, refresher or retraining using simulation [19], or utilizing mental practice [20] to maintain competency, is an essential starting point in moving toward this vision.

## Review

This scoping review was performed following the five-stage framework laid out by Arksey and O’Malley [21] and registered in the PROSPERO database (CRD420251005518). A database search was created in consultation with an expert librarian, and the search was performed in PubMed and then translated to CINAHL and EMBASE. PubMed, Embase, and CINAHL were searched using the following search strategy: the terms “emergency medicine," “emergency department," “trauma center*," “emergency service, hospital," and “physicians” were combined using the Boolean operator OR. The following additional terms: “skill(s) decay," “skill(s) retention," “skill(s) retain*," “skill(s) maintenance," “skill(s) maintain," “procedure(s) decay," “procedure(s) retention," “procedure(s) retain*," “procedure(s) maintenance," and “skill(s) maintain” were combined using the Boolean operator OR. The final search used the Boolean operator AND to search for relevant titles, combining the study population and the topic of interest. See Appendix 1 for the detailed search language used in each database.

Inclusion criteria

Our population of interest for this review is emergency medicine physicians, including attending as well as resident physicians, based on similar medical training and clinical practice. Studies that included interprofessional or interdisciplinary participants were included if a significant proportion of the study population were EM-trained physicians (either n > 9 or > 50% of the study population were EM physicians). This choice was made as an effort to include only studies most relevant to the practice of EM physicians while acknowledging value in the scholarship shared by other specialties and professions maintaining shared procedural competencies. Only studies that examined hands-on procedural and technical skills as described in the 2022 EM Model were considered for inclusion [22]. All contexts of assessment of skill decay were considered, including clinical practice or simulation-based training. Purely virtual assessments with no haptic component were not included. Relevant outcomes were evidence of skill decay or skill retention after any period of time.

Exclusion criteria

Because our research question was narrowly focused on emergency medicine physicians, studies were excluded if the population included exclusively laypersons; nurses; emergency medical technicians; paramedics; community health care workers; medical students; physicians not trained in EM, including anesthesia, internal medicine, surgery, pediatrics (without specifically stated additional pediatric EM training), obstetrics & gynecology, and surgical subspecialties. Studies examining non-technical skills, such as teamwork, leadership, communication, medical decision-making, and clinical reasoning, or any procedure not listed in the 2022 EM Model, were not included in this review. Studies that cited the concept of skill decay as background rationale without displaying evidence of skill retention or decay after a study intervention were excluded. Studies that examined the current frequency of procedural performance in a clinical setting and described implications for skill decay and need for ongoing training were not included if no evidence of skill decay or retention testing was included. Review articles, perspectives, and gray literature were not included in this scoping review.

Final search

Searches were performed in PubMed, CINAHL, and EMBASE in February 2025 using the terms and strategy detailed above. All resulting findings were uploaded into Covidence systematic and scoping review software (Veritas Health Innovation, Melbourne, Australia, [80]). All review articles that were unable to be included as primary sources in data extraction underwent reference searching using CitationChaser software ([81], 2021), and source articles were uploaded into Covidence [23]. After removal of duplicates, a total of 1349 articles were screened for inclusion criteria by title and abstract, and 172 articles underwent full-text review. Screening and reviewing were managed in Covidence, which was used to record all reviewer decisions. At each stage (i.e., abstract screening and full text review), inter-rater consistency of screening decisions was first established using ten randomly selected included articles with all raters screening the same articles, and conflicts being resolved through discussion and consensus among the team of reviewers. Subsequently, reviewers blindly and independently screened remaining articles for inclusion criteria. Articles were independently reviewed by two authors at each stage, with any additional conflicts being resolved through discussion and consensus among the team of reviewers.

Data extraction

An extraction table was created and piloted amongst all five reviewers. All reviewers performed independent data extraction of two randomly selected articles meeting the inclusion criteria. Extraction data from the two articles was reviewed for accuracy, and conflicts were resolved via discussion and consensus among the five reviewers. The custom data extraction form was then built into Covidence to record all extraction data, conflicts, and conflict resolutions. Reviewers independently and blindly extracted data for the remaining included articles. Pairs of authors independently extracted data in duplicate from each included study and later resolved any conflicts by consensus review.

Analysis

Descriptive content analysis of all publications meeting the inclusion criteria was performed. Because the goal of this review was to understand and describe the current state of the literature to inform and support future research rather than directly compare teaching interventions or assessment tools around procedural skills, we chose not to perform a formal quality assessment of included studies.

Results

**Figure 1:**
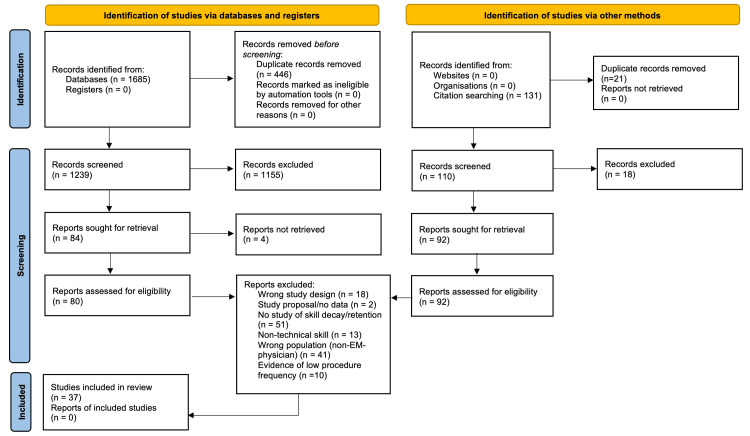
PRISMA flow chart

Overall, 37 articles were determined to meet the inclusion criteria (Figure 1) [24].

Out of the 37 articles that were determined to meet the inclusion criteria, eight were abstracts (22%). Most authors were from the United States (62%). Most studies were observational or quasi-experimental, while six (16%) were described as randomized controlled studies, and one was qualitative. The majority of studies took place at a single institution, and many had small sample sizes. Most studies were not blinded. 

The gold standard authors used to determine skill competency was heterogeneous and included expert opinion, skills checklists, global rating scores, and time to completion of the skills. Reporting of assessment tool validity evidence in articles was mixed; some articles utilized and cited previously validated and published assessment tools, while others created novel assessment tools and did not comment on validity. 

Included studies most often assessed Kirkpatrick level two outcomes of change in skills, knowledge, or attitudes (confidence). Seven articles (19%) also included level three outcomes by describing how often the new skill was performed in the clinical setting after initial training, while one study reported level four outcomes of how performing the procedure changed the management of the patient in clinical practice. 

See Table 1 for data extracted from included studies.

**Table 1:** Review articles meeting inclusion criteria ACLS: advanced cardiac life support, AHA: American Heart Association, BLS: basic life support, CME: continuing medical education, CPR: cardiopulmonary resuscitation, CVC: central venous catheter, DL: direct laryngoscopy/laryngoscope, DP: deliberate practice, ECMO: extracorporeal membrane oxygenation, ECPR: extracorporeal cardiopulmonary resuscitation, EM: emergency medicine, GRS: global rating scale, ISS: in situ simulation, LCC: lateral canthotomy & cantholysis, MCQ: multiple choice question, ML: mastery learning, NRP: neonatal resuscitation program, OCS: orbital compartment syndrome, OR: operating room, PALS: pediatric advanced life support, PEM: pediatric emergency medicine, PGY-#: post-graduate year - number of years, PIV: peripheral intravenous catheter, POCUS: point of care ultrasound, PTV: percutaneous transtracheal jet ventilation, SBE: simulation-based education, SBML: simulation-based mastery learning, SG: self-guided practice, US: ultrasound, USA: United States of America, VL: video laryngoscopy/laryngoscope, VR: virtual reality

Lead author, year	Country	Type of article	Procedural Skill Studied	Study Aims	Study Duration	Methodology	Study Participants	Sample size	Gold standard for competency	Assessment tool	Kirkpatrick Level Outcomes	Study conclusion	Length of time for retention or to decay
Ablordeppey, 2021 [25]	USA	Abstract	Ultrasound	Determine retention of TEE proficiency	12 months	Observational	EM Attending Physicians (academic faculty)	7	Ability to name, describe, and obtain 8 hands-on TEE images during the monthly examinations as determined via direct observation from the TEE expert.	7-8 months after training, participants asked to: recall the name of each TEE view, describe probe manipulation needed to achieve the view, successfully obtain each view on the TEE simulator	2 (skills, knowledge)	Focused, proficiency-based training on a TEE simulator results in durable skills retention after 6 months	Retention at 6 months
Ahn, 2016 [26]	USA	Original research	Intubation (VL)	(1) Establish a mastery-learning model for Glidescope VL intubation (2) Demonstrate the number of VL intubations required to establish mastery (3) Compare simulation-trained residents versus traditionally (clinical practice) trained residents	6 months	Randomized control trial	EM Residents (PGY1)	16	2 completely correct consecutive checklist performances for VL intubation	Checklist scores were developed using "rigorous step-by-step procedures, multiple sources of data, reviewed by board-certified EM physicians, & assessed for interrater reliability." Participant scores, times to completion, self-perceptions, and frequently omitted steps were compared between groups after initial training then at 3 and 6 months post-training	2 (skills)	Simulation-based mastery-learning resulted in enhanced with Glidescope VL skill that was resistant to decay over a 6-month period. Self-confidence and clinical experience were not adequate proxies for skill acquisition.	Retention at 6 months
Al Jadidi, 2023 [27]	Oman	Original research	CPR	Assess the effectiveness of a short BLS refresher training course 6-months after initial training on the retention of CPR-related psychomotor skills	12 months	Randomized control trial	Healthcare providers (physicians, nurses, paramedics) working in ED	38	5 components of high-quality CPR: compression rate (compressions/minute), depth (percentage), recoil (percentage), ventilation (percentage), and interruption time (seconds) per AHA ACLS recommended standards	Participants were assessed on CPR chest compression rate, depth, recoil, ventilation, and interruption time as recorded via QCPR monitoring at baseline, 6 months, and 12 months	2 (skills)	There was no skill decay at 6 months for either group, but the control group exhibited skill decay at 12 months when compared to intervention group	Retention at 6 months Skill decay at 12 months
Ander, 2004 [28]	USA	Original research	Airway Adjuncts	Assess the efficacy of a training program for EM residents in use of 3 rescue airway devices	12 months	Observational	EM Residents (3-year training program)	40	Time to ventilation (initial mouth opening to successful ventilation): LMA <30 sec, Fastrac <60 sec, Combitube <40 sec	Time to ventilation under direct observation in sim setting assessed immediately following teaching intervention and at unannounced re-testing at 6 & 12 months	2 (skills)	Emergency medicine residents can learn and retain airway adjunct skills with LMA, Fastrac, and Combitube	Retention at 12 months
Andreatta, 2015 [29]	USA	Original research	Intubation, Neonatal Resuscitation	(1) Establish validity evidence for competency assessment instruments (2) Evaluate the impact of live vs simulated feline airway training models on the abilities of clinicians to perform neonatal intubation	12 months	Quasi-experimental Design	Healthcare Providers (physicians [EM, PEM, PICU, NICU, anesthesia, burn trauma], nurses, residents, students, paramedics) that may be expected to perform neonatal intubation	294	46/46 points on semi-objective performance assessment instrument, 34/34 points on cognitive test	Scoring tool (created via literature review, 5 physician expert review, & construct validity testing) administered immediately after training then 18 weeks and 52 weeks after training	2 (skills, knowledge)	Simulation-based and live animal training models were relatively equivalent for the acquisition of neonatal intubation skills, though training with a simulation model showed higher retention scores.	Skill decay at 18 weeks (for live feline model group) Retention at 1 year in simulator group
Ansquer, 2019 [30]	France & Frech Guyana	Original research	Pediatric Resuscitation	Assess the impact of time elapsed since simulation based training on retention of pediatric emergency technical skills	5 years	Observational	Emergency Physicians (Residents & Attendings); PEM-trained physicians excluded	107	Maximum possible (100/100) score on Team Average Performance Assessment Scale (TAPAS)	Participants were videotaped and timed during a low-fidelity simulation scenario then scored on previously validated TAPAS scale. Participants also reported self-percieved stress rated on Stress-O-Meter (SOM) scale, and self-perceived confidence, satistaction, and realism on 0-10 Likert scale	1 (sim-related stress, satisfaction, realism) 2 (skills, confidence)	There was a decline in performance at 6-months after training and total loss of training benefit at 4 years after training.	Skill decay starting at 6 months, progressing to complete return to baseline at 4 years
Arntfield, 2015 [31]	Canada	Original research	Ultrasound	(1) Describe a TEE training protocol specifically tailored for the ED (2) Evaluate acquisition and retention of technical skills among EM physicians	6 weeks	Observational	Emergency Physicians (academic faculty and senior residents experienced in POCUS)	14	Adequate depiction of relevant anatomic features that typify each standard view and permit accurate interpretation as determined by 3 advanced echocardiographers (completed PTEeXAM certification training in TEE)	Simulator-based assessment immediately after training & 6 weeks post-training done by blinded video review of US images and scored acceptable/not acceptable by 3 experts. Time to complete the 4-view protocol recorded during assessments	2 (skills) 3 (rate of application of TEE skills in clinical practice)	EM physicians could successfully perform a focused TEE protocol after a 4 hour workshop and skills were retained after 6 weeks	Retention at 6 weeks
Chenkin, 2019 [32]	Canada	Abstract	Ultrasound	Determine the effectiveness of a TEE training workshop using a high-fidelity simulator	2 weeks	Observational	EM Residents	22	Successful views as determined by 2 blinded reviewers using an "anchored scoring tool"	"Anchored scoring tool," time to scan completion, and diagnostic accuracy used to assess performance immediately after training and 1-2 weeks after the training session	2 (skills)	The simulation-based workshop was effective for teaching EM residents the TEE protocol.	Retention at 1-2 weeks
Coggins, 2019 [33]	Australia	Original research	CPR	Assess retention of skills with mechanical CPR	6 months	Randomized control trial	Healthcare Providers (doctors, nurses) working in one tertiary academic ED setting	112	Lowest possible time to initiation of mechanical CPR; no errors on checklist created by consensus between investigators (unclear validation)	Time to initiate mechanical CPR recorded & error checklist scored by 2 investigators immediately after training and 6-months post-initial training	2 (skills)	After skill acquisition, a refresher simulation training at 4 months resulted in improved time to initiation for mechanical CPR.	Skill decay at 6 months (without refresher training)
Diaz, 2022 [34]	USA	Abstract	Ultrasound	Evaluate EM resident skill acquisition and retention of performing TEE after completion of a single simulation-based training session	2 months	Observational	EM Residents	18	Ability to obtain 3 TEE images of acceptible quality as determined by instructors	Adequately demonstrate 3 TEE views (dichotomous acceptible vs not acceptible) & time-tested on ability to recognize 3 separate simulated pathologies immediately, 4-week, and 8-weeks post training	2 (skills)	EM residents were able to perform a TEE protocol at 4 and 8 weeks post-training sessions.	Retention at 8 weeks
Douglass, 2013 [35]	Rwanda	Abstract	Ultrasound	Evaluate the effectiveness and sustainability of a POCUS training program for physicians based in hospitals in Rwanda	6 months	Observational	Physician Trainees	17	Score on image based assessment of 100%, score on observed structured clinical exam (OSCE) of 100%	Image based assessment pre-training, post-training & OSCE administered 4 times over 6 months	2 (skills, knowledge)	Initial training followed by periodic reinforcement of skills via supervised clinical scanning allowed trainees to learn and retain ultrasound knowledge and technical skills.	Retention at 6 months
Gillett, 2019 [36]	USA	Original research	Intubation (DL)	Identify how the frequency of intubation correlated with measured proficiency	3 months	Observational	Attending Emergency Physicians (24 general EM & 8 PEM-trained)	44	Successful completion of each item on 11-item intubation checklist with no struggle in performance (score of 10/10) as judged by pre-trained raters. Highest possible total score 21.	11-item intubation checklist (novel tool developed by authors using EM/anesthesia literature and expert opinion) & evaluation of overall psychomotor adeptness using a rating scale of 0-10 (0 representing significant “struggle” and 10 representing “no struggle.” The construct of “struggle” was defined by characteristics such as coordination, grace, dexterity, and timing)	N/A	Performing at least 3 or supervising at least 5 ETIs annually correlated with proficient performance on a skills assessment in study cohort.	Not described
Giorgetti, 2022 [37]	USA	Abstract	Ultrasound	Evaluate the effectiveness of a simulation workshop for TEE skill acquisition and retention	6 months	Observational	Emergency Physicians (Residents, Fellows, & Faculty)	16	Ability to perform TEE of comparable time and quality to cohort of board-certified cardiologists credentialed in TEE	Evaluation of image acquisition skills by performing 1 exam (12 images), followed by a series of 50 training exams (600 images) randomized and blinded for scoring. Retested at 1,3,6 month intervals post-training	2 (skills)	A 6-hour simulator-based TEE training workshop was effective.	Retention at 6 months
Gloria, 2011 [38]	Philippines	Abstract	CPR	Analyze BLS knowledge and skills acquisition and retention among EM residents	4 months	Observational	EM Residents	12	Perfect score on practical and written BLS examination	Practical and written examinations prior to training, immediately after, 1 week, 2 weeks, 1 month, and 4 months after the initial course	2 (skills, knowledge)	Skills practice may have to be repeated as soon as 1 week after the initial BLS training.	Skill decay at 1 week
Homan, 1994 [39]	USA	Original research	Thoracostomy	Evaluate the efficacy of an emergency-procedure laboratory in the development and retention of procedural skills	18 days	Observational	EM Residents (PGY1) & medical students	12	Successful (correct placement within the thoracic cavity, as indicated by tube fogging) insertion using proper technique in less than one minute	Dichotomous success or failure of tube thoracostomy plus time to completion of procedure of all attempts during initial trianing and 18-days post-training	2 (skills)	Procedure laboratories are effective for teaching tube thoracostomy psychomotor skills and in enhancing skill retention. An emphasis on repetition is essential for the improvement and retention of skills.	Retention at 18 days
Huang, 2024 [40]	Taiwan	Original research	Thoracentesis (Ultrasound-guided), Pericardiocentesis (Ultrasound-guided)	Evaluate the performance of novices after training with handmade phantoms for US-guided thoracocentesis and pericardiocentesis	3 months	Observational	Residents (PGY1 from various specialties) & UGY6 medical students	50	Assessment forms developed based on a literature review and expert consensus	Assessment performed independently by 2 instructors not involved in enrollment/training; one on-site and the other evaluating videos with novices’ faces masked. Score on assessment form, puncture time, (from the initiation of the attempt to fluid aspiration), number of puncture attempts all recorded. Self-reported pre- and post- curriculum confidence levels recorded. 3 month re-evaluation on commercial high-fidelity task trainer performed.	1 (course satisfaction) 2 (skills, confidence)	Novices displayed improved performance in thoracentesis during the 3-month assessment compared to the immediate assessment. ]Novices demonstrated performance comparable to that of experienced residents in thoracocentesis. Novice performance in pericardiocentesis did not reach a similar level and even declined.	Retention at 3 months for thoracentesis Skill decay at 3 months for pericardiocentesis
Kei, 2023 [41]	USA	Original research	Intubation (DL & VL)	Compare resident versus attending physician intubation time, success, accuracy, Cormack-Lehane (CL) grades obtained, and subjective ease of the intubation across various laryngoscopy devices	Not specified	Observational	Emergency Physicians (Residents PGY1-3 & Attendings)	50	Confirmation of intubation and accuracy as measured by having a single experienced emergency physician visualize the ETT position markers in relation to the vocal cords using VL after each intubation attempt	6 intubations per physician (standardized equipment and mannequin, randomized order of techniques). Each trial timed and recorded by a single experienced emergency physician (start: pick up blade, end: cuff up plus blade out). Participant-rated Cormakc-Lehane classification, participant-rated ease of intubation.	N/A	When using a GlideScope hyperangulated blade, resident physicians overall intubated faster and more accurately than attending physicians. There was increased time needed for DL intubation in PGY-3 versus PGY-2 residents	Skill decay at about 1 year for DL
Laack, 2014 [42]	USA	Original research	Central Venous Access (Ultrasound-guided)	(1) Determine if central line training intervention results in sustained improvement in central line placement (2) Determine variability in performance after training	6 months	Observational	EM Residents (PGY1-3)	26	15/15 (composite score 1.0) dichotomously scored items on Central Venous Catheterization Proficiency Scale, validated, previously confirmed high interrater agreement	Blinded investigators scored video review of trainee performance using CVC Proficiency Scale Composite scores pre-training, immediately post-training, 3 months post-training. Procedure performance was timed.	2 (skills)	CVC line placement improved after the training. Composite scores decreased in the 3 months after training (ie. performance quality decreased), but remained higher than baseline	Skill decay at 3 months
Lin-Martore, 2021 [43]	USA	Original research	Ultrasound	Develop an internet-based curriculum for POCUS for intussusception and longitudinally assess its efficacy by assessing technical skill, knowledge, and confidence of PEM providers	3 months	Observational	PEM faculty & fellow physicians	17	22/22 on skill checklist (created after literature review and consensus of 3 PEM US experts & one pediatric radiologist) as scored by single observer; 100% on MCQ knowledge test	Direct observation of POCUS performed on a standardized patient using 22-point checklist. Knowledge acquisition assessed via online MCQ. Confidence assessed via survey instrument using a 5-point Likert scale	2 (skills, knowledge, confidence) 3 (POCUS exams performed in clinical setting)	PEM physicians showed improvement in knowledge, confidence, and technical after the curriculum, all of which were maintained after 3 months.	Retention at 3 months
Lin-Martore, 2021 [44]	USA	Original research	Not specified	Explore: "(1) How do PEM physicians conceptualize maintenance of procedural skills? (2) What motivates PEM physicians to maintain procedural skills? (3) What are barriers to procedural skill maintenance?"	6 months	Qualitative	Academic PEM Physicians	12	N/A	Thematic analysis of semistructured interview transcripts	N/A	Although there was no clear definition of "procedural skill maintenance" among participants, they described an overall motivatation to maintain procedural skills. Participants described feeling unease with certain procedures, desire for more trianing, and emphasized the importance of autonomy, accessibility, and choice in successful training interventions. Multiple barriers to ongoing training were described and participants felt that more support from institutions and leadership would be needed to overcome these barriers.	Not described
Nazerian, 2020 [45]	Italy	Original research	Ultrasound	Assess EM physicians's ability to obtain and maintain skills in performing TEE after clinical training in the cardiac surgery OR	12 weeks	Observational	Emergency Physicians (Residents & Attendings)	10	Score of 5 in probe insertion, view aquisitions, and overall performance as subjectively rated by anesthesiologist tutor. Rating system (from 1 to 5) as follows: 1=inadequate, 2=insufficient, 3=sufficient, 4=good and 5=excellent	Skills assessed by anesthesiologist (scale 1-5) at the end of OR-training and 12 weeks after the completion of training. A study observer in the ED recorded time from ED patients' arrival to TEE; clinical consequences of ED-performed TEE during 12-weeks post-training period recorded.	2 (skills) 3 (performance of TEE skills in clinical setting) 4 (documented changes to patient clinical care: identified dissection, tamponade, corrected cardiac compression site, vascular cannulation guidance)	2 hours of lecture and 8 hours of hands-on clinical training in a cardiac surgery OR led to acquisition and maintenance of TEE skills in EM physicians at 12 weeks.	Retention at 12 weeks
Osei-Ampofo, 2018 [46]	Ghana	Original research	Ultrasound	Assess ultrasound skill and knowledge retention as well as perceived impact on local practice 9-11 months after initial training	11 months	Observational	EM Residents	20	100% correct on written knowledge test and a score of at least 70% on OSCE	Written tests ans OSCEs performed immediately after training and 9-11 months after training. Written test included 6 vignettes of case presentations and US clips. OSCE evaluated POCUS exams for lungs, cardiac views, IVC, FAST, aorta, DVT. An additional electronic survey was sent to study participants and one-on-one interviews were conducted.	1 (reactions) 2 (skills, knowledge) 3 (interviews of how POCUS being applied clinically)	Long-term knowledge and skill retention were demonstrated after the teaching intervention.	Retention at 9-11 months
Pelletier, 2024 [47]	USA	Original research	Lateral Canthotomy	Describe experiential learning intervention to teach management of orbital compartment syndrome (OCS) and have learners demonstrate proficiency in lateral canthotomy & cantholysis (LCC)	3 months	Observational	EM Residents (PGY1-4)	18	100% (19/19) on skills checklist (developed via modified Delphi process) as scored by 2 study team members	Lateral canthotomy & cantholysis procedure checklist, MCQ test, Likert self-efficacy-scale (1-5) completed pre-, immediately post-, and 3 months post-training	1 (course evaluation/reactions) 2 (skills, knowledge)	Training participants demonstrated significantly higher checklist-based performance scores at 3 month retention testing	Retention at 3 months
Petrosoniak, 2023 [48]	Canada	Original research	Surgical Airway (bougie-assisted cricothyrotomy)	Compare strategies of deliberate practice plus mastery learning (DP+ML) versus self-guided (SG) practice for performance of a bougie-assisted cricothyrotomy	12 months	Randomized control trial	EM Residents	176	Maximum GRS score 35. No agreed upon standard in the literature for BAC performance time nor is there evidence for what constitutes clinically significant differences in performance time when comparing techniques or training methods	Skills assessed by 3 trained, independent, blinded airway experts using 7-item validated instrument (possible scores 7-35) of pre-training, immediately post-training, and 6-12 months post-training video-recorded performance. Speed of performance (first palpation of neck to successful ventilation) also recorded.	2 (skills)	Both DP+ML and SG practice strategies led to skill improvement compared to baseline with subsequent decline in skills over time. DP+ ML training perticipants performed the skill more efficiently during the retention testing.	Skill decay at 6-12 months
Pirie, 2023 [49]	Canada	Original research	Intubation, Intraosseous Line Placement, Surgical Airway, Thoracostomy, Ultrasound, Pediatric Resuscitation	Evaluate a mandatory simulation-based competency based medical education program for PEM faculty of both common and “high-acuity, low-opportunity” emergency procedures, including POCUS	2 years	Observational	PEM Faculty & Pediatric ED Nurses	88	Meets or exceeds minimal procedure checklist items & GRS score of at least 3 on previously validated 5-point Objective Score of Technical Skills (OSAT)	Task-specific checklists (designed by expert opinion & consensus among PEM faculty, general surgery faculty, RT education lead w/ minimum competency established a priori) & GRS (OSAT) for each procedure station recorded for participants at single point in time during the training. Team Emergency Assessment Measure (TEAM) scores recorded during 88 unannounced in situ team simulations after introducing teaching intervention compared to ISS score pre-intervention.	1 (coure evaluation) 2 (skills) 3 (perfomance during in situ sim)	Most participants demonstrated procedural skill competence on checklists. TEAM scores during in situ simulations improved or were maintained over time.	Retention in team in situ performance at 1 year (not specified for individual procedure skills)
Saggar, 2024 [50]	USA	Original research	Wound Management (tourniquet application)	Compare the retention of tourniquet placement skills between in-person virtual reality (VR) training to the current standard of in-person lecture training	3 months	Observational	EM Residents (PGY1-4)	53	Stepwise application of tourniquet placement for an actively hemorrhaging wound, as agreed upon by the nationally recognized guidelines from the American College of Trauma Surgeons and Stop the Bleed campaign in under 90 seconds	Observation of tourniquet application at days 0 and 90 by trained, blinded evaluators (board-certified EM physicians) using the standardized, National Registry Hemorrhage Control Skills Lab rubric: New York State (NYS EMT) tourniquet skills rubric	2 (skills)	No significant difference in successful tourniquet placement by EM residents when trained via in-person lecture vs VR, which suggests VR may be a useful adjunct to traditional in-person medical training for tourniquet placement.	Retention at 3 months
Schmitz, 2021 [51]	USA	Original research	CPR	Determine whether timing of last ACLS/BLS training impacts skills performance of EM physicians	Not specified	Observational	Emergency Physicians (Residents & Attendings)	113	Participants passed the scenario if they completed all critical actions (assess responsiveness, breathing, circulation, call for help) and delivered 2 cycles of high-quality CPR (100-120 compressions/min, >2 inches, full chest recoil, chest rise w breaths) with a ratio of 30 compressions: 2 rescue breaths per the RQI skills check requirements	Simulation case performed at EM conference, each participants' performance assessed at single point in time	2 (skills)	No difference in high-quality CPR delivery between EM physicians who recently underwent BLS training and those who did not, including EM physicians who had not taken a BLS course in over 2 years.	Retention up to 2 years
Schott, 2021 [52]	USA	Original research	Ultrasound	Assess ultrasound knowledge, skills, and frequency of clinical use 6-9 months after participating in a brief POCUS training course	21 months	Observational	Physicians (18% EM)	127	100 points (maximal score) on the unique ultrasound skill rating scale, 100% (30/30) score on knowledge test	Online 30-question knowledge test (developed by consensus of VA POCUS faculty & investigators) and directly observed skills test on 100-point rating scale (developed by authors, unclear validation) conducted by faculty preceptor were assessed pre-training, immediately post-training in person, and once 6-9 months after training using teleUS software for retention.	1 (course evaluation) 2 (skills, confidence) 3 (frequency of POCUS use in clinical practice)	Practicing physicians can retain POCUS skills 8 months after training, regardless of frequency of POCUS use after training.	Retention at 6-9 months (median 8)
Shrestha, 2023 [53]	Nepal	Original research	CPR, Defibrillation	Assess junior doctors' retention of advanced life support knowledge and technical skills in management of shockable cardiac arrest	6 months	Observational	Residents	43	Score of at least 90% on 25-action checklist informed by UK Resuscitation Council guidance on shockable cardiac arrest	Knowledge assessment survey & simulation-based skills checklist as assessed by one EM physician pre-training, immediate post-training, 30 days post, and 60 days post.	2 (skills, knowledge)	Technical skills required for management of cardiac arrest declined at 30 days, while knowledge declined at 60 days.	Skill decay at 30 days
Stross, 1983 [54]	USA	Original research	CPR	Examine retention of knowledge and skills related to ACLS and to determine the effectiveness of two techniques (readings or patient cases) for reinforcing learning and improving retention of cognitive & motor skills	18 months	Randomized control trial	Physicians (35% EM)	132	Criteria for the successful completion of each skill as defined by the AHA	All participants received a pre-test to identify a series of 12 dysrhythmia strips, perform one- and two-person CPR, and initiate appropriate therapy in simulation cases. This was repeated 1-year after training.	2 (skills, knowledge)	ACLS motor skills were not retained after one year.	Skill decay at 1 year
Tan, 2018 [55]	USA	Original research	Thoracostomy	Compare a simulation task trainer with a cadaveric model for teaching chest tube insertion	9 months	Quasi-experimental Design	Residents (50% EM PGY1-2)	16	All 15 critical action criteria met on previously published mastery learning checklist	Checklist score determined via direct observation by same faculty member that conducted initial mastery training checklist at 7 months post-taining. Pre-training and post-training self-reported confidence levels were assessed via questionnaires. Participants maintained clinical procedure logs during the period from initial training to retention testing.	2 (skills, confidence) 3 (chest tubes logged during clinical practice)	Both simulation models (task trainer and cadaver) were associated with significantly increased confidence in chest tube placement. There was no difference in skills between groups.	Retention at 7 months
Turner, 2023 [56]	USA	Original research	Surgical Airway (cricothyrotomy)	Establish a minimum number of simulated cricothyrotomy attempts to reach proficiency	3 years	Observational	EM Residents (PGY1-3)	69	Successful placement was confirmed through direct visualization of the 3D-printed model	Speed of successful airway placement timed in person or via video review for each participant. Repeated attempts recorded in PGY1 year, PGY2 year, PGY3 year. Participant self-reported confidence recorded	2 (skills, confidence)	Participants reached a plateua of procedural proficiency after six practice attempts at cricothyrotomy using a simulated model. This is more than the current ACGME recommendation of 3 cricothyroidotomies. Periodic retraining was important to maintain skills.	Slight skill decay compared to prior best performance at 1 year with initial attempt but fewer practice repetitions needed to meet then excede prior performance
VanDyck, 2018 [57]	USA	Abstract	Extracorporeal membrane oxygenation (ECMO)	Evaluate a simulation course designed to train ED physicians and nurses to initiate ECPR within 30 minutes	> 3 months	Observational	Emergency Physicians & Nurses	5 teams (3 ED physicians, 3 ED nurses)	Full ECPR support (3 L/min) established within 30 min of simulated patient arrival & all critical action checklist items completed successfully without safety violations	Proportion of runs in which full ECPR support initiated within 30 minutes of simulation start, time to cannulation, time to prime circuit, time to initiate ECPR all recorded (not stated if direct observation vs video review). Critical action checklist used to score performance. Scenario monitored for safety violations.	2 (skills)	The 2-day course utilizing high-fidelity simulation did result in ED physicians and nurses being able to rapidly initiate ECPR during a cardiac arrest scenario. These skills were retained for at least 3 months.	Retention at 3 months
Werner, 2016 [58]	USA	Original research	Central Venous Access (Ultrasound-guided)	Assess how an educational intervention using simulation-based mastery learning affects the competency of PEM physicians performing US-guided femoral CVC placement	12 months	Observational	PEM-trained Attending Physicians	28	Competency was defined as successfully performing all 7 critical steps on the checklist	Direct-observation checklist developed and validated using a modified Delphi method and checked for interrater reliability. Participants were scored pre-intervention, immediately after, and then 2 and 12 months after the intervention (in situ).	2 (skills)	The intervention resulted in improved US-guided CVC placement and that this improvement was maintained over 12 months.	Retention at 12 months
Whitman, 2012 [59]	USA	Abstract	Surgical Airway (percutaneous transtracheal ventilation)	Assess EM residents' ability to learn and retain knowledge & skills performing percutaneous transtracheal jet ventilation	2 months	Observational	EM Residents (PGY1-3)	15	Survey score (100%) & self-reported confidence 5/5 on Likert scale	Survey of self-reported knowledge, skills, confidence prior to training, immediately after, and 2 months after training	2 (confidence, knowledge, self-assessed skills)	Simulated training using an animal model lead to increased skills and confidence in performing PTV with good retention at 2 months	Retention at 2 months
Whitmore, 2019 [60]	USA	Original research	Extracorporeal membrane oxygenation (ECMO)	Test whether EM physicians and nurses can acquire and retain skills to initiate ECPR using high fidelity simulation training	3 months	Observational	Emergency Physicians & Nurses	7 teams (3 ED physicians, 3 ED nurses)	Full ECPR support (3 L/min) established within 30 min of simulated patient arrival & all critical action checklist items completed successfully without safety violations	Proportion of simulations in which full ECPR support achieved within 30 min on videotaped performance assessed before training, immediately after, and 3-month post-training. Time to: complete cannulation, prime circuit, full ECPR support achieved were recorded at each assessment. Scores on critical action scoring sheet & incidence of safety violations recorderd at each assessment.	2 (skills)	A high fidelity ECPR simulation training course enables teams of emergency medicine physicians and nurses to acquire and retain the skills necessary to rapidly and safely initiate ECPR in a high-fidelity simulation scenario.	Retention at 3 months
Yamamoto, 2023 [61]	Japan	Original research	Intubation	Compare first-year residents' skill retention in video laryngoscopy (VL) versus direct laryngoscopy (DL) endotracheal intubation	1 year	Randomized control trial (cluster)	Residents (PGY1)	46	Failure defined as tube not passed through glottis within 60 sec. Success was anything not meeting definition of failure. POGO (percent of glottic opening) scores recorded as secondary outcome	Time (when resident first touched mannequin to tip of ETT through glottis) required to successful intubation & POGO scores (as visually evaluated by faculty member) measured on SimManVR 3G immediately post-rotation, 3 and 6 months post-rotation	2 (skills)	Intubation training with video laryngoscopy shortened intubation procedure time and showed improved long-term skill retention compared to traditional direct laryngoscopy.	Retention at 6 months

Using descriptive content analysis of all articles meeting inclusion criteria, five key themes were identified: (1) limited focus on skill decay as a primary outcome, (2) short and variable timeframes of assessment, (3) limited representation of procedural diversity, (4) heavy reliance on simulation and checklist-based assessments, (5) sparse data on attending physicians and community-based practice.

Limited Focus on Skill Decay as a Primary Outcome

Most studies examined procedural skill retention only as a secondary outcome measure within broader evaluations of training programs and curricular innovations. Thirty of the included studies (81%) used skill retention as an outcome measure to assess the impact of a teaching intervention, comparing pre-intervention assessment, immediate post-intervention assessment, and then retention testing at one or more timepoints post-intervention. Most articles did not provide a rationale for the decision to perform the retention assessment at a certain time point. Five studies (13.5%) measured skill performance at one point in time and determined the correlation between physician performance quality and time elapsed from prior training or the correlation of performance quality with the frequency of procedures performed. Few studies were explicitly designed to isolate and analyze skill decay as a primary outcome. 

Short and Variable Timeframes of Assessment

Study duration and time interval of skills assessments were variable, with most studies assessing retention or decay at 2-12 months, though retention testing occurred as early as one week after initial training. Only two of the included studies (5.5%) described skill retention or decay beyond a one-year timeframe. Most studies assessed skills at fixed intervals, not waiting until actual decay was detected. These timeframes appear driven primarily by study feasibility rather than evidence-based reasoning regarding when decay is most likely to occur. Very few studies attempted to track performance until observable decay emerged, which constrains our understanding of the natural course of technical skill degradation over time. This gap in longitudinal tracking limits the generalizability of existing literature to inform the length of the skills warranty once mastery is achieved and the frequency that refresher training is needed. Additionally, when considering skill decay along the larger learning curve [2], there are confounding variables such as quality of initial learning or overlearning that may impact the rate of skill decay in different clinicians. The number of variables affecting learning and forgetting curves may help explain why this has been challenging to study.

Limited Representation of Procedural Diversity

Overall, 43% of all included studies involved ultrasound; eleven looked at image acquisition skills, and five looked at procedural guidance applications, including central venous access, ECPR, thoracentesis, and pericardiocentesis. Since point-of-care ultrasound is a relatively new EM procedural competency skillset, the frequent inclusion of retention testing as an outcome measure in these articles may be seen as an encouraging trend and a reflection of the increasing attention on skill decay in medical education literature. Studies of cardiopulmonary resuscitation (CPR) skills also appeared frequently in this review, representing 30% of included articles. An additional 27% involved airway procedures, including endotracheal intubation, surgical airway, and airway adjunct device placement. CPR and airway skills are fundamental life-saving skills for which maintenance of competency is crucial, making it relatively unsurprising that there have been efforts made to understand decay in these specific skills. “Resuscitation” is listed within the procedural skills in the EM Model, but notably, application of these skills in the clinical environment is often complex, involving the co-application of technical and non-technical skills. We selected studies that examined the procedural skills involved in resuscitation, but one could argue that the non-technical skills involved in application may be just as important in improving patient outcomes as technical skills maintenance. 

Several ACGME-required EM procedures, including cardiac pacing, lumbar puncture, orthopedic reductions, procedural sedation, and vaginal delivery, are not represented in any included studies, suggesting ready opportunities for future research of these essential skills. Additional HALO procedures, such as resuscitative hysterotomy, thoracotomy, and escharotomy, were also not represented in the included articles. The limited range of procedures studied may be due to challenges in simulation-based task trainer fidelity for certain procedures, access to training resources, logistics around organizing training and retesting, or lack of reliable externally validated instruments. 

Heavy Reliance on Simulation and Checklist-Based Assessments

Nearly all studies used simulation-based technologies, including task trainers, mannequins, OSCE/standardized patients, in situ simulation, or virtual reality in either training or retention testing. Most studies utilized simulation and checklists or global rating scales (GRS) to assess competence. While these tools offer structured, reproducible metrics, their real-world correlation with clinical performance remains unclear, and a possible lack of transfer from the simulation environment to clinical practice was often cited by article authors as a potential limitation. Although there is existing evidence that simulation-based mastery learning results in improved patient outcomes for central venous access [62], further research that clarifies the relationship between simulation-based performance and patient outcomes would help support simulation as a methodology to achieve as well as maintain mastery. 

Sparse Data on Attending EM Physicians and Community-Based Practice

About half of the included articles, 49%, had study populations of exclusively trainees, while only five articles (13.5%) reported a study population of exclusively attending EM physicians. Several articles (11%) reported exclusively PEM-trained study participants. Nearly all included studies were conducted in academic EM programs, limiting generalizability to broader EM practice environments, especially community-based or rural settings. Within this largely academic group, attending physicians were underrepresented as study subjects, despite concerns about skills decay occurring among clinicians who supervise more often than perform procedures. A previous study of community versus academic EM procedures found that community practice attending physicians performed more intubations and tube thoracostomies, while attending physicians at tertiary centers performed more ultrasound procedures, indicating different practice settings may predispose to different rates of skill decay in different procedures [63]. The literature does contain published survey data of attending physicians who reported feeling effects of skills decay [4,64,65], and several articles have described the implementation of procedure training curricula for continuing professional development to prevent decay [66-69]. However, none of these published surveys or curricula were found to assess skill decay or retention over time and, although informative, do not help answer questions around the minimum frequency of training or number of procedures necessary to maintain competency over a given period of time.

Future directions

Future studies should focus on skill decay as a primary outcome, track skill performance until measurable decay is observed, and ideally, expand beyond simulation-based metrics to include real-world clinical outcomes, where feasible. How different methods of refresher training outside of clinical practice (e.g., low-fidelity task trainers, high-fidelity simulations, mental rehearsal, extended reality, podcasts, or video reviews) affect the rate of skill decay or ideal frequency of refresher training is also not fully understood in the context of EM physicians [20,70-77]. To understand the experience and training needs of emergency physicians with varying practice patterns, future studies should include more diverse practice settings, more attending physician experience, and multi-institutional data to enhance the generalizability of findings. Evaluating the role of procedural supervision in mitigating skill decay may be a particularly relevant question in academic settings. Agreement on a gold standard of procedural competency beyond the current ACGME-required procedure counts, including development and utilization of standardized, validated assessment tools for these procedures, could enhance consistency in training and comparability across studies. In addition to developing more accurate, reliable, and broadly applied scoring instruments, incorporating new technology into the assessment of skill decay, like hand-motion analysis, may be useful as an objective tool for assessing skill decay over time [78,79].

Limitations

Gray literature was not included in this scoping review, as our research question aimed at reviewing the existing evidence base for time to skill decay in EM. Additionally, publications that only examined the frequency of procedures performed in clinical settings and then inferred that skill decay is likely to occur based on infrequent performance were not included in this review [6-17].

We focused our review on articles with study populations of emergency medicine attending physicians or resident doctors because we felt the clinical practice environment of emergency medicine, with a lack of predictability and infrequent opportunities for practice of some procedures, may have a unique effect on skill decay compared to other medical specialties. Because initial skill learning may affect the rate of decay, we limited the studies to only those with a population who had received a Doctor of Medicine or equivalent degree. Although these choices helped us answer our narrower research question, there is a potential that informative studies from other medical specialties or health professions were not captured in this review. We included studies from outside the United States to increase the generalizability of findings, though it is unclear if differences in medical training systems between countries impacted study findings at all.

Because the aim of this scoping review was to perform descriptive analysis, and such heterogeneity among studies was encountered in the process, formal comparative analysis, quality assessment, and risk of bias assessment were not performed. However, we did note that many included studies were limited by small sample size, single site, and nonrandomized or nonblinded study design. Many authors describe the risk of selection bias or confounding from participant learning outside of the intended study design. There was a low frequency of patient-centered clinical outcomes described, and many studies noted a potential limitation of a possible lack of transfer between simulation and clinical practice.

## Conclusions

Procedural skill decay is a complex process that depends on several variables: procedure type, initial learning, experience level of the clinician, and frequency of practice. Overall, this scoping review reveals a literature base that is heterogeneous in design, limited in scope, and still evolving. While there is growing recognition that technical skill decay is a relevant concern in EM, most current studies offer only partial answers. More rigorous, longitudinal, and generalizable research is required to establish clear, evidence-based practices for maintaining procedural competency across the continuum of emergency physician training and practice.

## Appendices

Appendix 1: database searches

PubMed Search

("emergency medicine" or "emergency medicine"[mesh] or "emergency department" or "emergency service, hospital"[Mesh] or "trauma center*" or "physicians"[mesh]) and ("skill decay"[title/abstract:~5] or "skills decay"[title/abstract:~5] or "skill maintenance"[title/abstract:~5] or "skills maintenance"[title/abstract:~5] or "skill maintain"[title/abstract:~5] or "skills maintain"[title/abstract:~5] or "skill retention"[title/abstract:~5] or "skills retention"[title/abstract:~5] or deskilling or "skill retaining"[title/abstract:~5] or "skills retaining"[title/abstract:~5] or "skill retain"[title/abstract:~5] or "skills retain"[title/abstract:~5] or "procedure decay"[title/abstract:~5] or "procedures decay"[title/abstract:~5] or "procedure maintenance"[title/abstract:~5] or "procedures maintenance"[title/abstract:~5] or "procedure maintain"[title/abstract:~5] or "procedures maintain"[title/abstract:~5] or "procedure retention"[title/abstract:~5] or "procedures retention"[title/abstract:~5] or "procedure retain"[title/abstract:~5] or "procedures retain"[title/abstract:~5])

CINAHL Search

(emergency medicine or emergency department or trauma center or (MH "emergency medicine") or (MH “physicians, emergency”) or (MH "emergency service")) and (skill N5 decay OR skills N5 decay OR skill N5 maintain OR skills N5 maintain OR skill N5 maintenance OR skills N5 maintenance OR skill N5 retain OR skills N5 retain OR skill N5 retention OR skills N5 retention OR skill N5 fade OR skills N5 fade OR deskilling OR procedure N5 decay OR procedures N5 decay OR procedure N5 maintain OR procedures N5 maintain OR procedure N5 maintenance OR procedures N5 maintenance OR procedure N5 retention OR procedures N5 retention OR procedure N5 retain OR procedures N5 retain)

EMBASE Search

(emergency medicine or ‘emergency medicine’/exp or emergency department or 'emergency ward'/exp or 'emergency health service'/exp or 'emergency physician'/exp) and (skill near/5 decay or skills near/5 decay or skill near/5 maintenance or skills near/5 maintenance or skill near/5 maintain or skills near/5 maintain or skill near/5 retention or skills near/5 retention or deskilling or skill near/5 retaining or skills near/5 retaining or skill near/5 retain or skills near/5 retain or procedure near/5 decay or procedures near/5 decay or procedure near/5 maintenance or procedures near/5 maintenance or procedure near/5 maintain or procedures near/5 maintain or procedure near/5 retention or procedures near/5 retention or procedure near/5 retain or procedures near/5 retain)
